# Supplementary material for: Urokinase-Type Plasminogen Activator Receptor (uPAR) Cooperates with Mutated KRAS in Regulating Cellular Plasticity and Gemcitabine Response in Pancreatic Adenocarcinomas
Source: Cancers (Basel). 2023 Mar 3;15(5):1587. doi: 10.3390/cancers15051587 (PMC10000455; doi:10.3390/cancers15051587)

WBs to Figure 2c

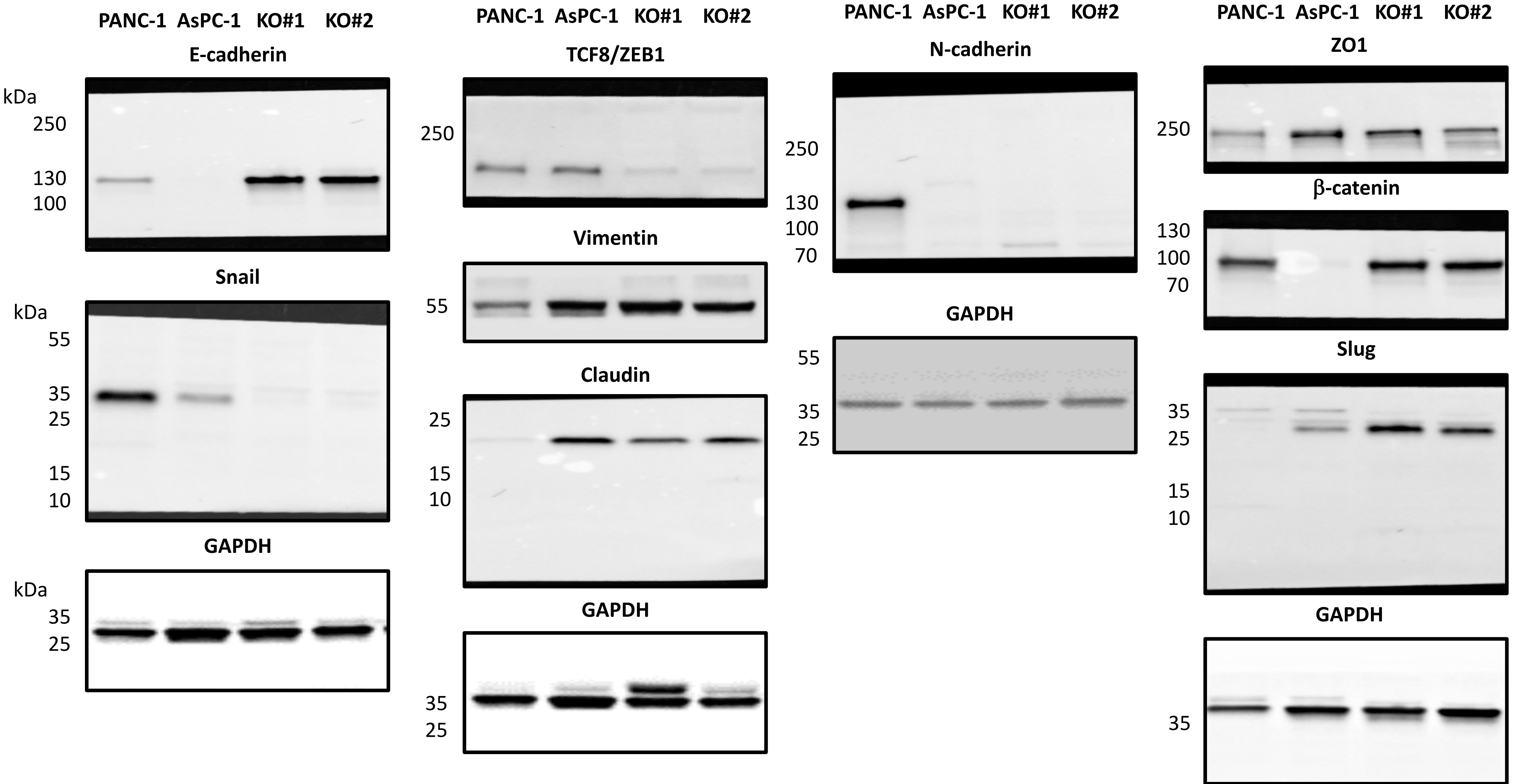

WBs to Figure 3a

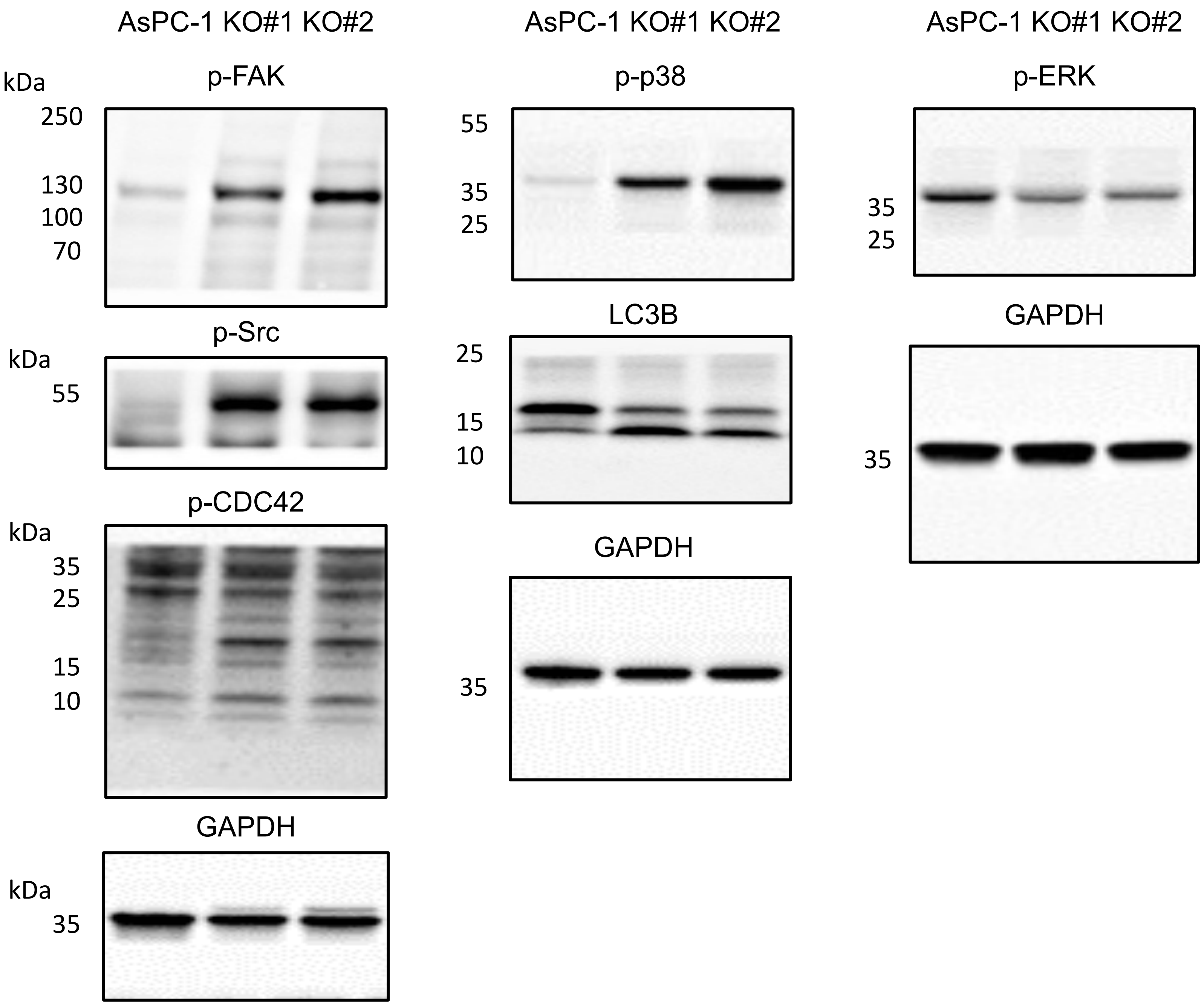

WBs to Figure 3b

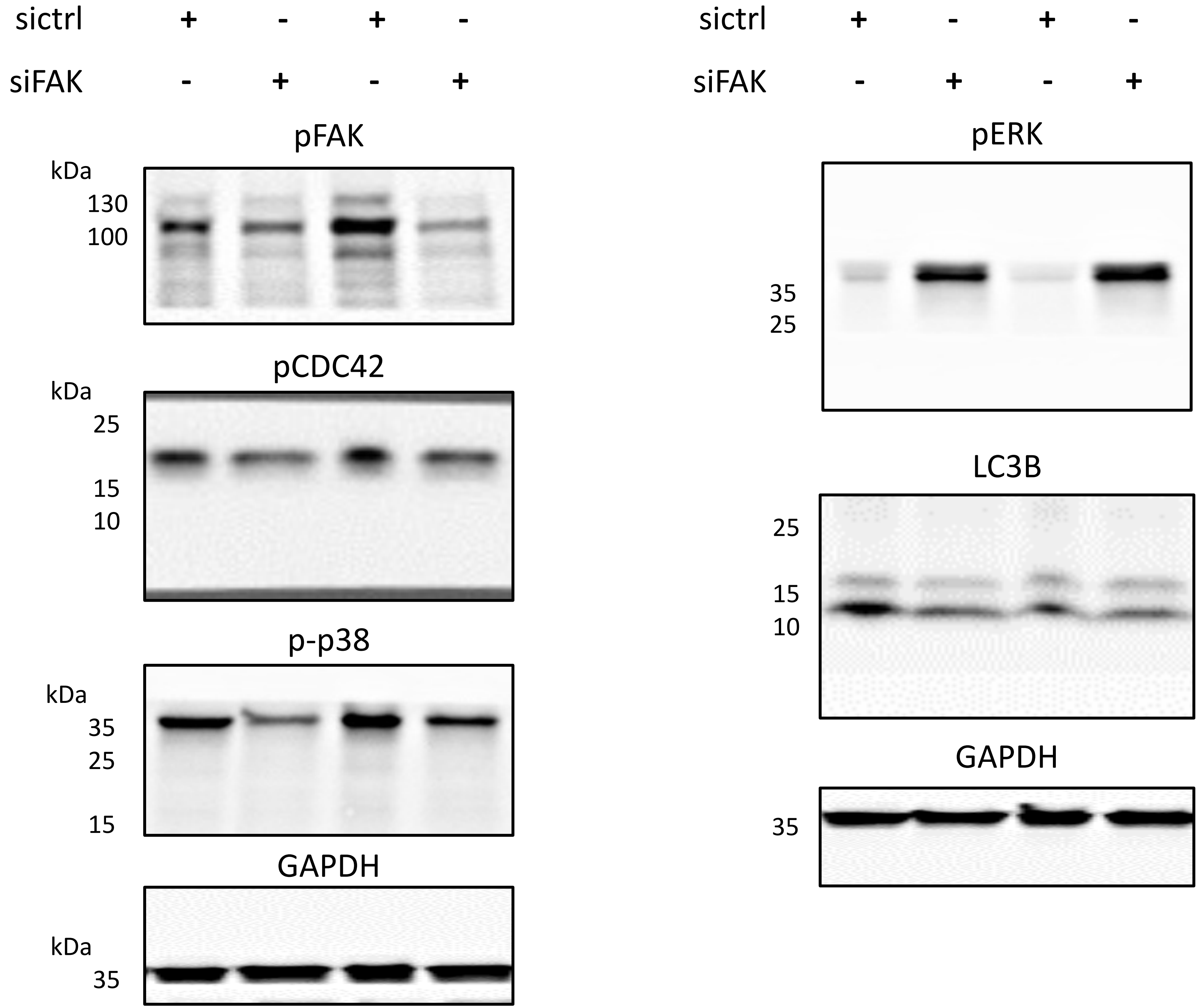

WBs to Figure 3d

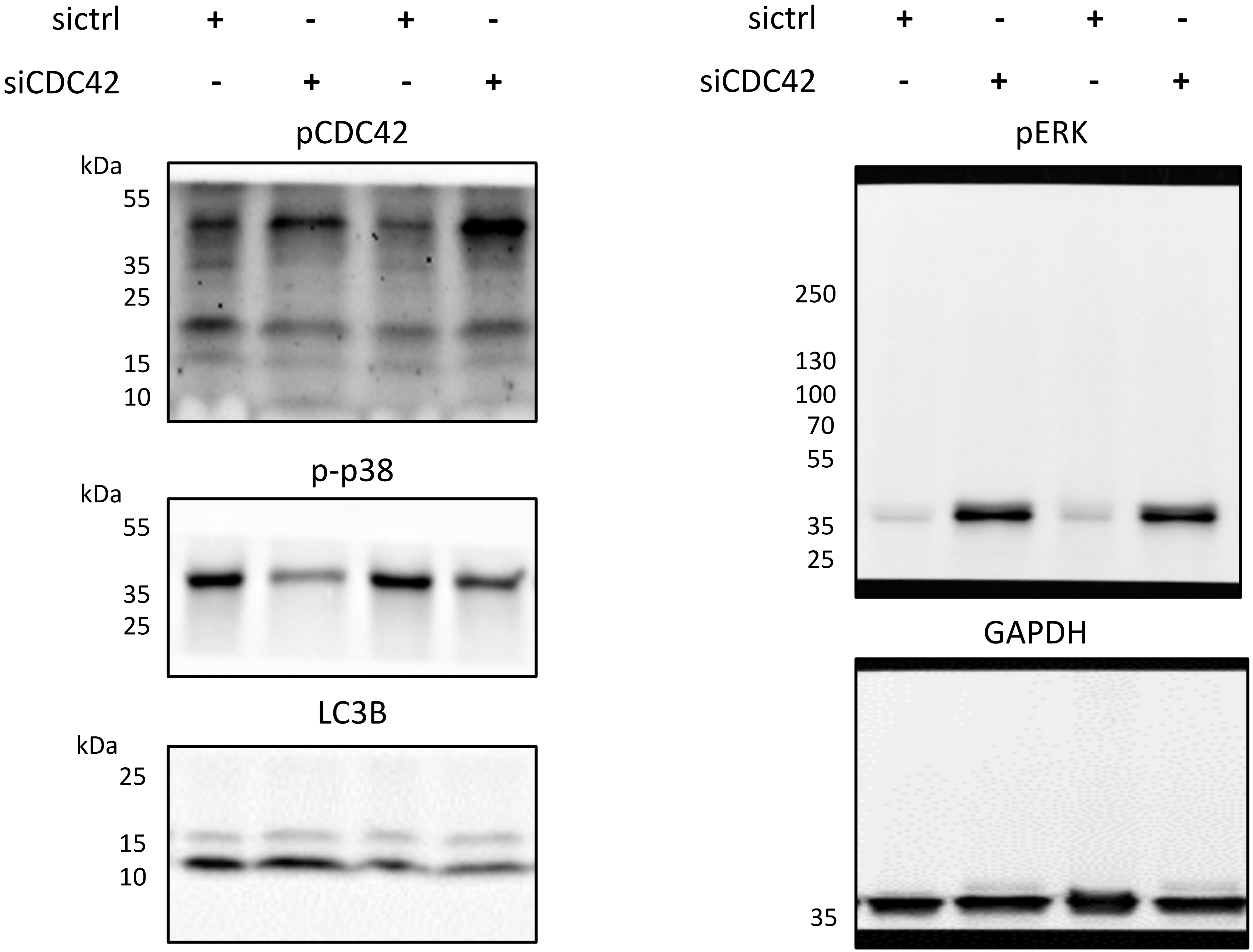

WBs to Figure 3f

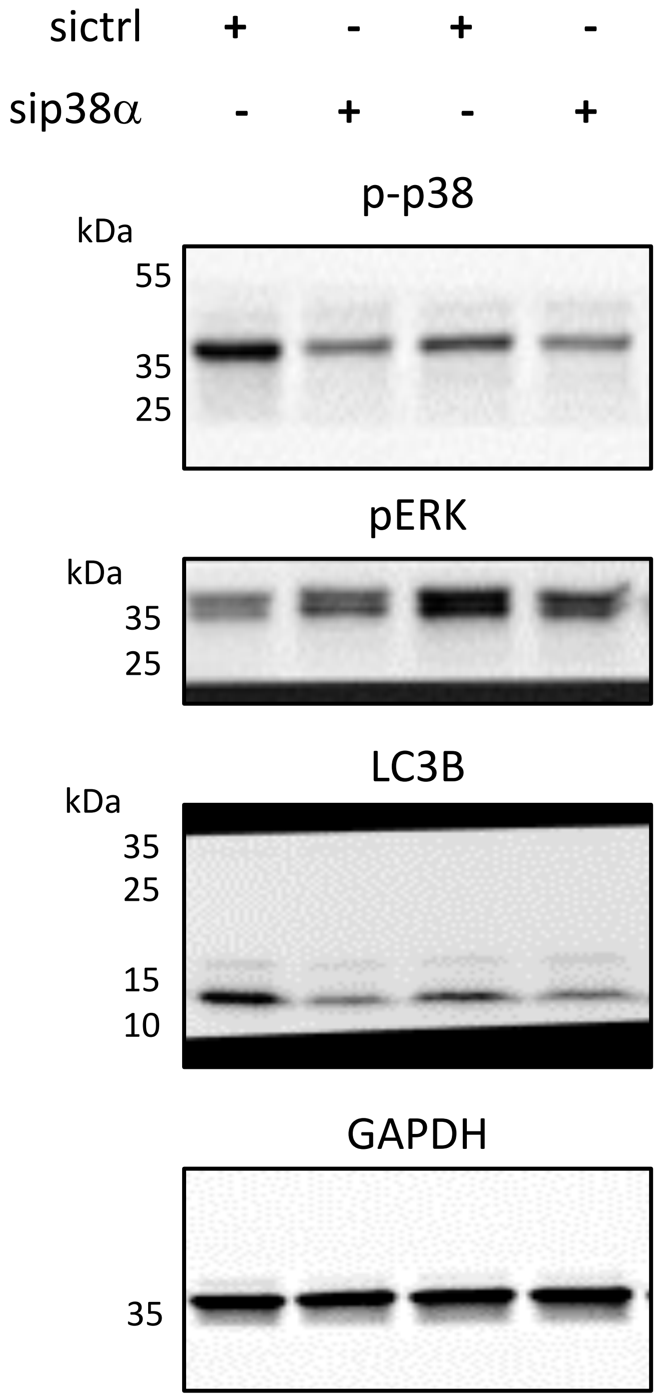

WBs to Figure 4a

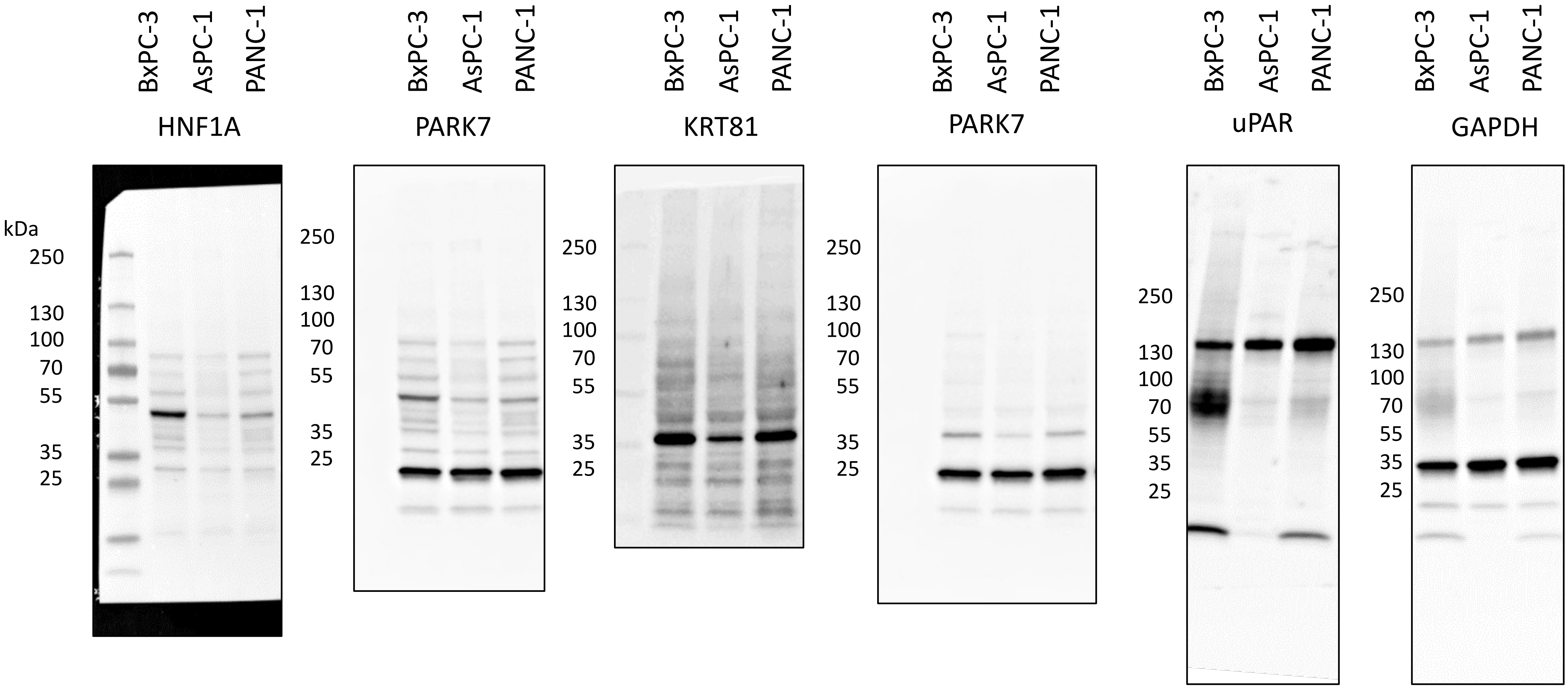

WBs to Figure 4e

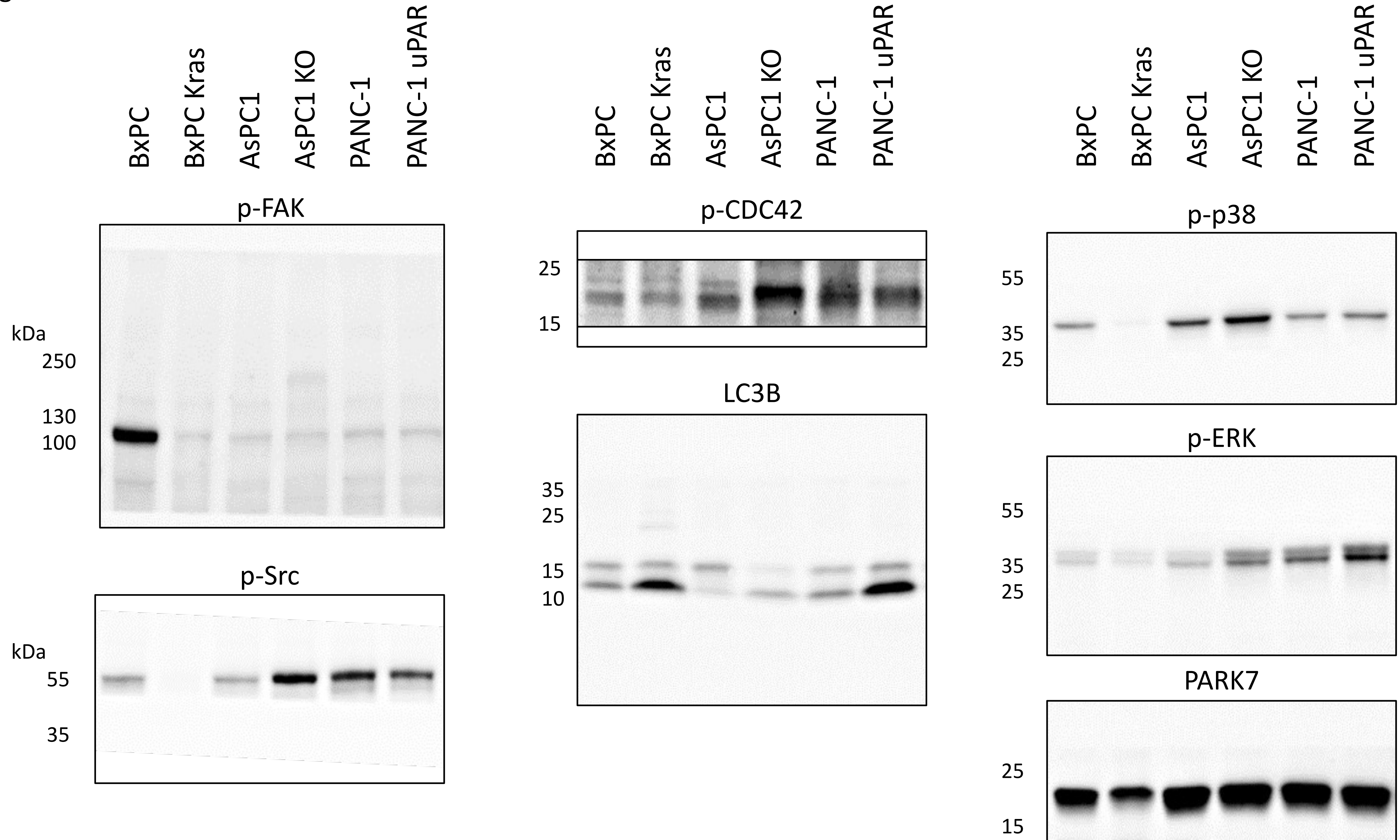

Supplement: Supplementary file 1 [file cancers-15-01587-s001.zip › cancers-2217766- Supplementary Material File S1 Uncropped WB images.pdf]
