# Supplementary material for: Urokinase-Type Plasminogen Activator Receptor (uPAR) Cooperates with Mutated KRAS in Regulating Cellular Plasticity and Gemcitabine Response in Pancreatic Adenocarcinomas
Source: Cancers (Basel). 2023 Mar 3;15(5):1587. doi: 10.3390/cancers15051587 (PMC10000455; doi:10.3390/cancers15051587)
Supplement: Supplementary file 1 [file cancers-15-01587-s001.zip › cancers-2217766-supplementary.pdf]

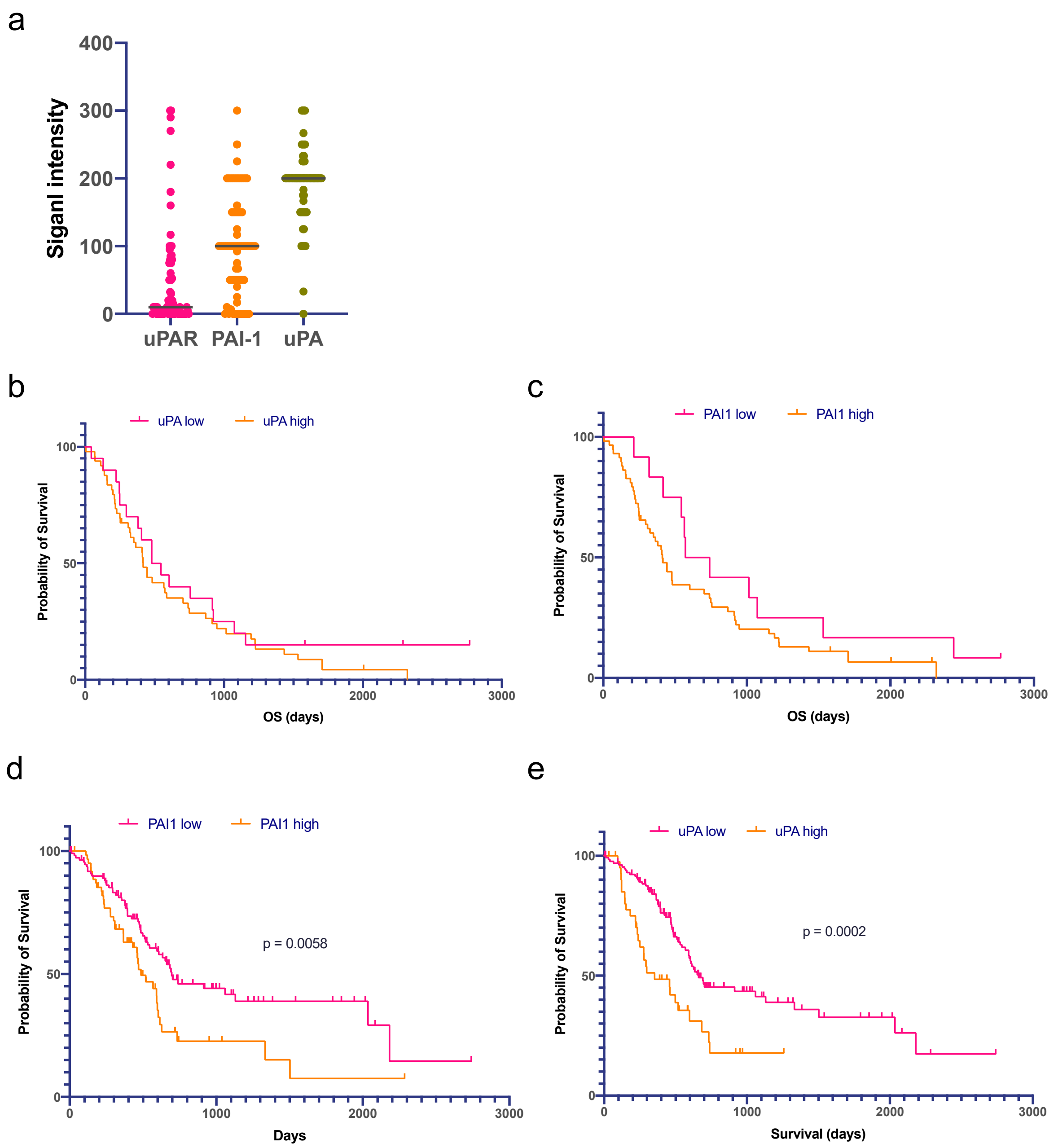

Supplementary Figure S1: (a) Staining intensities (300 score) of uPA, uPAR, and PAI1 of 69 PDAC patient samples. (b) OS analysis of PDAC patients with low vs. high protein expression of uPA on immunohistochemistry, and (c) PAI1. (d) OS analysis of PDAC patients with low vs. high mRNA expression of uPA (Cox-Mantel-test, *P* = 0.0475) and (e) PAI1.

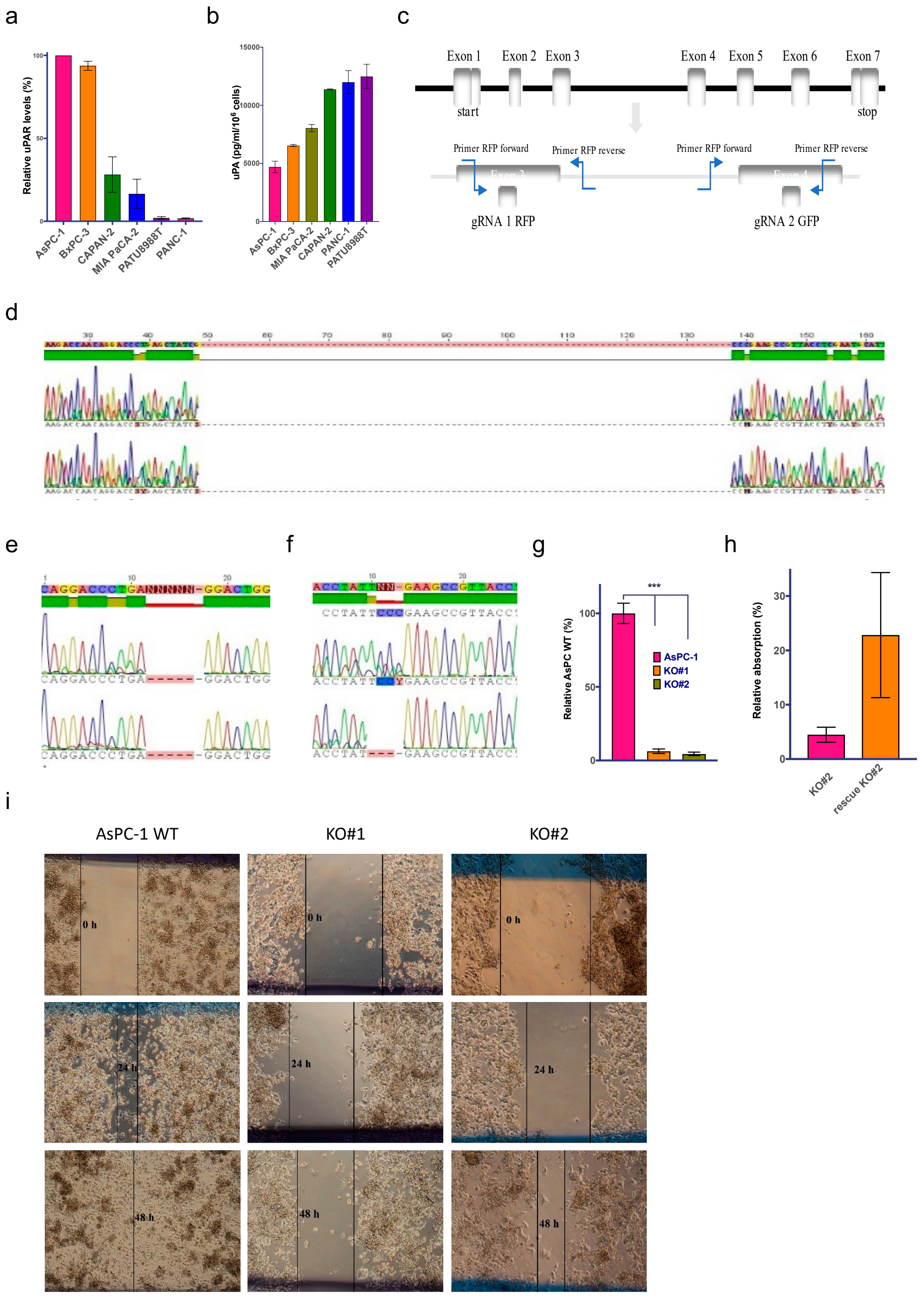

Supplementary Figure S2: (a) uPAR and (b) uPA protein levels of the pancreatic cell lines AsPC-1, BxPC-3, CAPAN-2, MIA PaCa-2, PATU8988T, and PANC-1 measured by ELISA. (c) Schematic representation of the *uPAR* CRISPR/Cas9 strategy. Two gRNAs were directed against exon three and exon 4 of the *uPAR* gene and were used to generate *uPAR*$^{-/-}$ clones. (d–f) Sanger sequencing analysis of two *uPAR*$^{-/-}$ clones consisting of a large deletion and a site-specific mutation. (g) ELISA measurement of uPAR levels in KO#1 and KO#2 compared to uPAR WT and (h) of rescue KO#2 by re-expressing uPAR compared to KO#2. (i) Exemplary pictures of the migration assay of AsPC-1 WT, KO#1 and KO#2 over 48 hours.

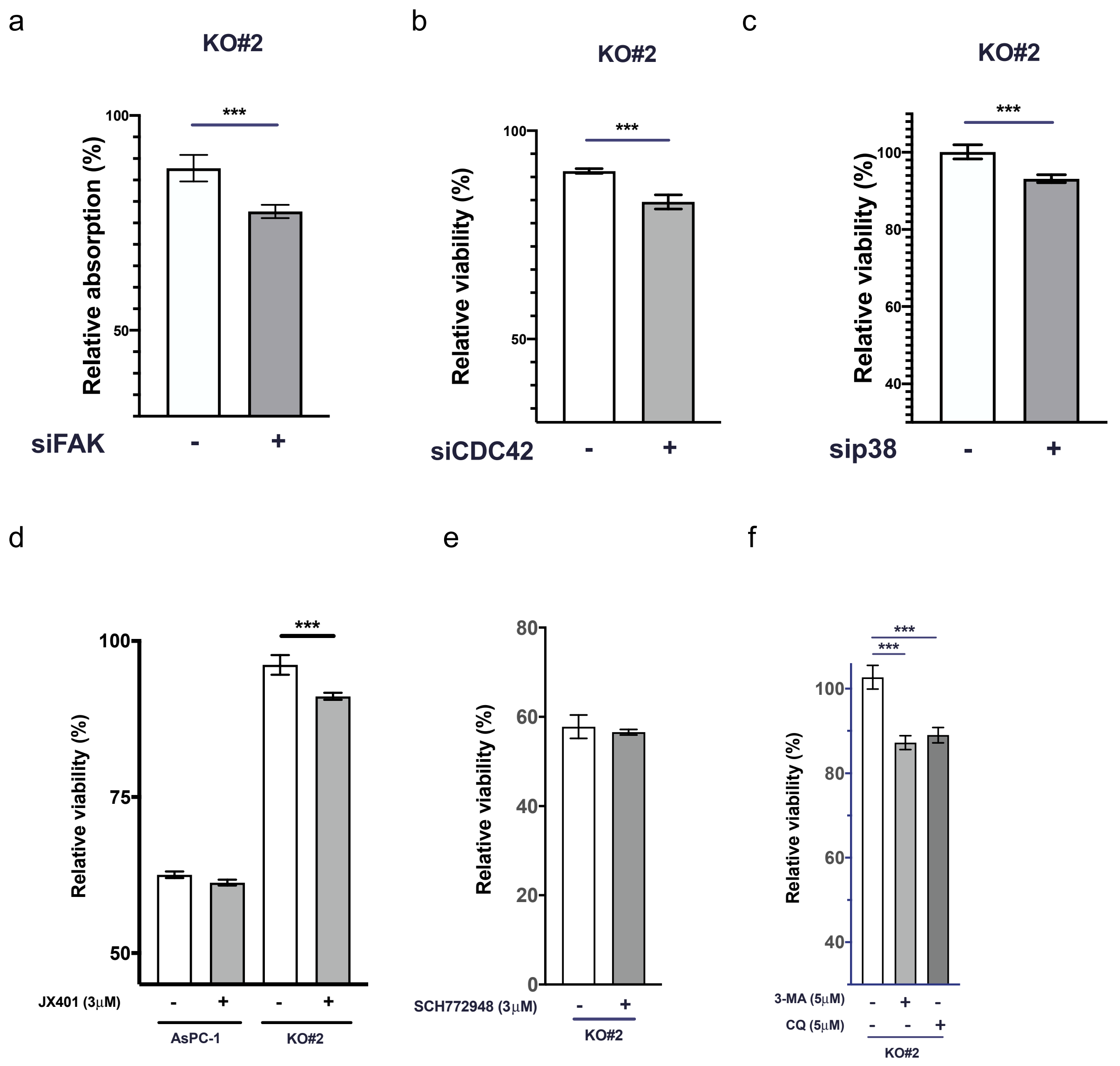

Supplementary Figure S3: Gemcitabine treatment after siRNA knockdown and p38 inhibition in AsPC1 WT and *uPAR$^{-/-}$* cells. Gemcitabine response (0.1 µM, 72h) after siRNA knockdown (80nM, 24h) of (a) FAK, (b) CDC42 and (c) of p38 in AsPC1 KO#2. (d) Gemcitabine treatment of AsPC-1 WT and *uPAR$^{-/-}$* cells (KO#2) in combination with the p38 inhibitor JX401. (e) Gemcitabine treatment (0.1 µM, 72h) vs. combination with ERK inhibition (SCH772948, 3µM) of *uPAR$^{-/-}$* cells (KO#2). (f) Treatment of uPAR knock-out clones (KO#2) with either gemcitabine (0.1 µM) or in combination with the autophagy inhibitors 3-MA (5 µM) or CQ (5 µM) (n = 4).

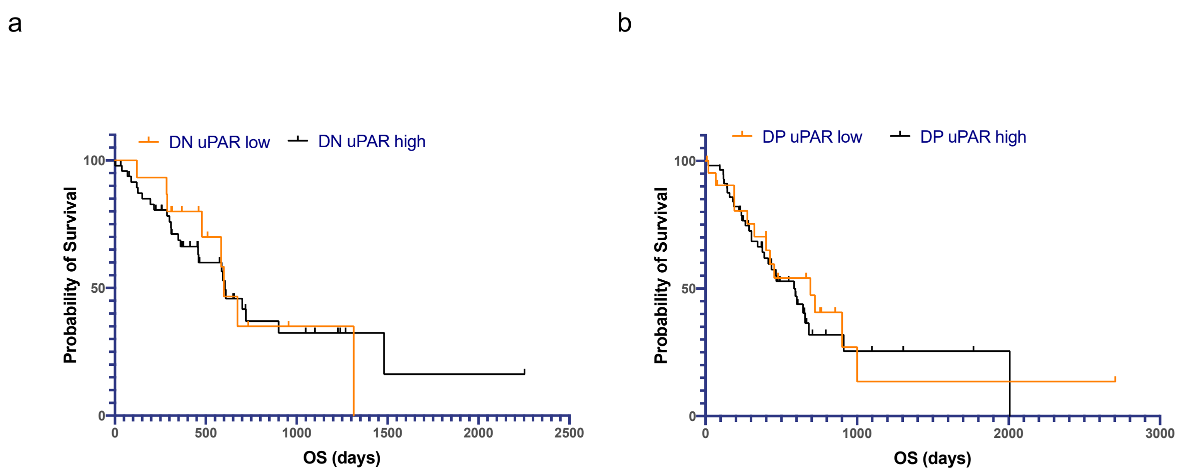

Supplementary Figure S4: Kaplan Meyer OS analysis of TCGA patient cohort. (a) uPAR low (n=15) vs uPAR high (n=49) in DN cases and (b) uPAR low (n=22) vs uPAR high (n=69) in DP cases.

Table S1: Human PDAC cell lines with TP53 and KRAS mutation status

Cell line	p53 status	cDNA	Protein	KRAS status	cDNA	Protein
AsPC-1	mut/mut	c.403delT	p.C135fs*35	mut/mut	c.35G>A	p.G12D
CAPAN-2	mut/mut	c.375G>T	p.I255N	mut/mut	c.35G>T	p.G12V
MIA PaCa-2	mut/mut	c.742C>T	p.R248W	mut/mut	c.34G>T	p.G12C
PANC-1	mut/mut	c.818G>A	p.R273H	mut/mut	c.35G>A	p.G12D
PATU8988T	mut/mut	c.844C>T	p.R282W	mut/mut	c.35G>T	p.G12V
BxPC-3	mut/mut	c.659A>G	p.Y220C	wt/wt		

Table S2: siRNAs (Qiagen)

Target	Name	Cat. No.
KRAS 1	Hs_KRAS2_8	SI02662051
KRAS 2	Hs_KRAS2_3	SI00071015
p38MAPK 1	Hs_MAPK14_5	SI00300769
p38MAPK 2	Hs_MAPK14_7	SI00605164
FAK 1	Hs_PTK2_5	SI00287791
FAK 2	Hs_PTK2_9	SI00301532
CDC42 1	Hs_CDC42_7	SI02757328
CDC42 2	Hs_CDC42_15	SI04381671

Table S3: Antibodies and chemicals.

Primary Antibodies

Gene name	Antibody name	Company	Dilution, Concentration	Method
uPA	ATN291	Prof A.P. Mazar	0.5 µg/ml	IHC
uPAR	ATN617	Prof A.P. Mazar	1.2 µg/ml	IHC
PAI1	13801-1-AP	ProteinTech	0.8 µg/ml	IHC
phospho-ERK1/2, Thr202/Tyr204	#4370	Cell Signaling	1/1000	WB
phospho-p38 MAPK, Thr180/Tyr182	#4511	Cell Signaling	1/1000	WB
phospho-FAK, Tyr397	#8556	Cell Signaling	1/1000	WB
phospho-SRC, Ser17	#12432	Cell Signaling	1/1000	WB
phospho-Rac1/CDC42, Ser71	#2461	Cell Signaling	1/1000	WB
SQSTM1/p62	#8025	Cell Signaling	1/1000	WB
LC3B	#3868	Cell Signaling	1/1000	WB
Vimentin	#5741	Cell Signaling	1/1000	WB
Claudin-1	#13255	Cell Signaling	1/1000	WB
N-Cadherin	#13116	Cell Signaling	1/1000	WB
β-Catenin	#8480	Cell Signaling	1/1000	WB
ZO-1	#8193	Cell Signaling	1/1000	WB
TCF8/ZEB1	#3396	Cell Signaling	1/1000	WB
E-Cadherin	#3195	Cell Signaling	1/1000	WB
GAPDH	#5174	Cell Signaling	1/1000	WB
PARK7	ab18257	Abcam	1/1000	WB
beta-actin	clone AC-74	Sigma-Aldrich	1/1000	WB
KRAS	MBS168791-5	Biolabs	1/1000	WB
Ki67	GA626	Agilent	1/1000	WB
HNF1A	sc-393925	Santa Cruz	1/200, 1/1000	IHC/WB
KRT81	sc-100929	Santa Cruz	1/200, 1/1000	IHC/WB

Secondary Antibodies

Gene name	Antibody name	Company	Dilution, Concentration	Method
Polyclonal rabbit anti-mouse immunoglobulin/HRP	2024-02	Sigma-Aldrich	1/1000	WB
polyclonal goat anti-rabbit immunoglobulins/HRP	2025-08-31	Sigma-Aldrich	1/1000	WB
Envision anti-mouse	K4001	Agilent	ready to use	IHC
Envision anti-rabbit	K4003	Agilent	ready to use	IHC

Chemicals

Name	Article number	Company
Gemcitabine	G5423	Sigma-Aldrich
Chloroquine (QC)	C6628	Sigma-Aldrich
SCH772984	S7107	Selleckchem
3-MA	CAY13242	Biomol
JX401	2657	Bio-techne
